# Drug interactions between ALK inhibitors and warfarin with concurrent use of bucolome: a case report

**DOI:** 10.1186/s40780-023-00282-1

**Published:** 2023-05-01

**Authors:** Takashi Kato, Yusuke Kunimoto, Manabu Kitagawa, Yuichiro Asai, Tomoko Kimyo, Hiromasa Nakata, Mamoru Takahashi, Hirofumi Chiba, Hiroki Takahashi, Atsushi Miyamoto, Masahide Fukudo

**Affiliations:** 1 Department of Pharmacy, Sapporo Medical University Hospital, South-1, West-16, Chuo-Ku, Sapporo, 060-8543 Japan; 2 Department of Respiratory Medicine and Allergology, Sapporo Medical University School of Medicine, Sapporo, 060-8556 Japan; 3 Division of Pharmaceutical Health Care and Sciences, Sapporo Medical University School of Medicine, Sapporo, 060-8556 Japan

**Keywords:** Alectinib, Crizotinib, Ceritinib, Warfarin, Bucolome, International normalized ratio (INR), Drug Interaction Probability Scale (DIPS)

## Abstract

**Background:**

Alectinib, crizotinib, and ceritinib, are anaplastic lymphoma kinase-tyrosine kinase inhibitors (ALK-TKIs) that exhibit high protein binding, and their metabolism is associated with the cytochrome P450 (CYP) isoenzymes 2C9 or 3A4. The plasma protein binding rate of warfarin, which is used to prevent and treat venous thromboembolism, is also high. Warfarin is a racemate of S-warfarin and R-warfarin, which are metabolized by CYP2C9 and CYP3A4, respectively. Reports on the drug interactions between each of the above-mentioned ALK-TKIs and warfarin with concurrent use of bucolome are currently lacking.

Case presentation.

We report a case of a patient receiving warfarin and bucolome, whose international normalized ratio (INR) increased after sequential treatment with alectinib, crizotinib, and ceritinib. The patient was a 61-year-old man with a history of aortic valve regurgitation, who was receiving warfarin treatment following aortic valve replacement. Bucolome, which can enhance the effect of warfarin, was also used simultaneously. The patient was diagnosed with primary lung adenocarcinoma, and *ALK* rearrangement was detected during second-line chemotherapy. After progression of the disease with chemotherapy, sequential treatment with alectinib, crizotinib, and ceritinib was initiated. Pretreatment INR values were in the therapeutic range (target INR of 2–3) but increased to supratherapeutic levels each time after initiation of alectinib, crizotinib, or ceritinib treatment. Adjustment of warfarin dose or discontinuation of bucolome were necessary to maintain the therapeutic INR range. There were no serious bleeding events or substantial changes in dietary intake.

Displacement of plasma protein binding or competitive inhibition of metabolism by alectinib, crizotinib, and ceritinib could increase the plasma concentration of the unbound form of warfarin, resulting in high INR values. In addition, alectinib, crizotinib, and ceritinib might cause displacement of bucolome from plasma proteins, followed by displacement of warfarin or inhibition of warfarin metabolism caused by the unbound form of bucolome.

**Conclusions:**

Close monitoring of INR and adjustment of warfarin dosage are needed during treatment with alectinib, crizotinib, or ceritinib in patients who receive warfarin with concurrent use of bucolome.

**Supplementary Information:**

The online version contains supplementary material available at 10.1186/s40780-023-00282-1.

## Background

Recently, considerable progress has been made in the treatment of anaplastic lymphoma kinase (ALK)-rearranged non–small-cell lung cancer (NSCLC) with additional ALK-tyrosine kinase inhibitors (TKIs) [1–4]. ALK-TKIs include alectinib, crizotinib, and ceritinib, all of which are available in Japan and have some common pharmacokinetic characteristics. First, the plasma protein binding rates of alectinib, crizotinib, and ceritinib are greater than 99%, 91%, and 97.2%, respectively; thus, they all show high protein binding. Second, their metabolism is associated with the cytochrome P450 (CYP) isoenzymes 2C9 and 3A4, which play an important role in drug metabolism. Alectinib is primarily metabolized by CYP3A4, crizotinib is metabolized by CYP3A4/5 and is a time-dependent inhibitor of CYP3A, and ceritinib is a substrate of CYP3A and may reversibly inhibit CYP2A6, CYP2C9, and CYP3A. Moreover, ceritinib may inhibit CYP3A in a time-dependent manner. Therefore, drug interactions may occur when these drugs are used concomitantly with other drugs.

The dosage of warfarin, which is used to prevent and treat venous thromboembolism, is adjusted to achieve a target international normalized ratio (INR). The variability in the efficacy of warfarin caused by drug interactions must also be carefully considered. The rate of warfarin binding to blood proteins is > 90%. Warfarin is a racemate of S-warfarin and R-warfarin, with a distribution volume of 0.14 L/kg, each. S-warfarin is mainly metabolized by CYP2C9, and R-warfarin is metabolized by CYP3A4, CYP1A2, and other isozymes. Statements on drug interactions between warfarin and alectinib or crizotinib are not available in the prescription information for these drugs. Although some studies have reported on the drug interactions between warfarin and each of the above-mentioned ALK-TKIs [5–7], INR elevation during treatment with alectinib, crizotinib, or ceritinib concurrent use of warfarin and bucolome has not been reported. Here, we report the first case of a patient receiving warfarin and bucolome whose INR increased after sequential treatment with alectinib, crizotinib, and ceritinib.

## Case presentation

A 61-year-old man was diagnosed with primary lung adenocarcinoma (pT1bN1M0, stage IIA) by intraoperative pathological diagnosis, and underwent partial resection of the left lower lobe of the lung and lymph node dissection. The patient had a history of aortic valve regurgitation and was receiving warfarin treatment to prevent thromboembolic complications following aortic valve replacement at the age of 35 (target INR of 2–3).

**Fig. 1:**
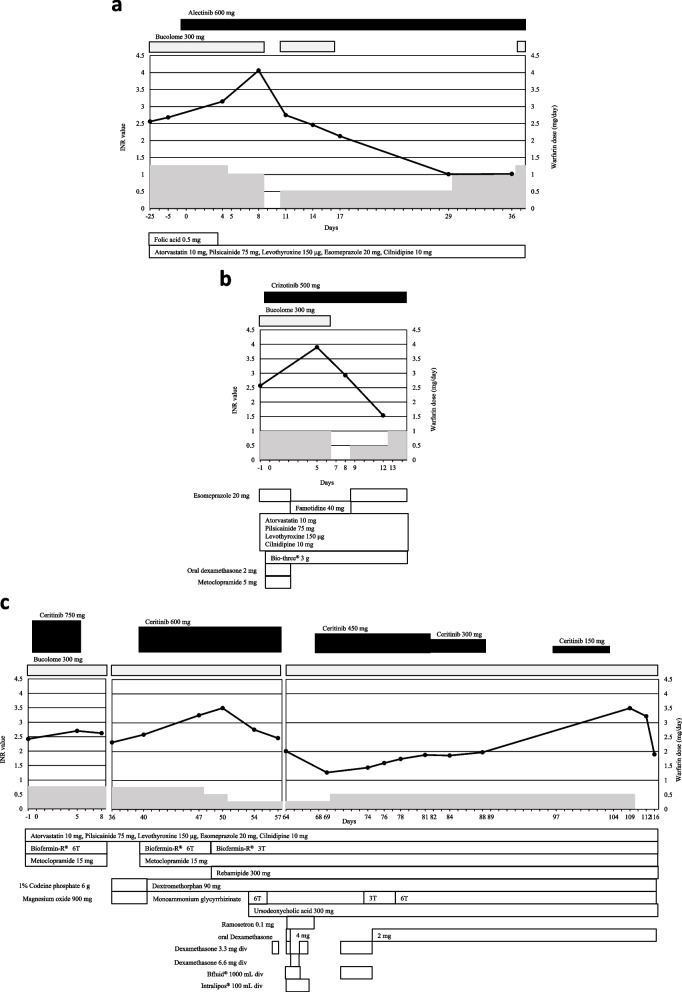
The patient’s INR values and daily doses of warfarin and bucolome during treatment with alectinib (**a**), crizotinib (**b**), and ceritinib (**c**). INR: international normalized ratio.div: drip intravenous

The patient received adjuvant chemotherapy in 2011; however, recurrence of lung cancer was confirmed in 2016, and *ALK* rearrangement was detected during pemetrexed-based second-line chemotherapy. Therefore, treatment with oral alectinib was initiated at a dose of 300 mg twice daily (day 0, Fig. 1a). The patient’s oral medications on admission included folic acid 0.5 mg daily, atorvastatin 10 mg daily, pilsicainide 75 mg daily, levothyroxine 150 μg daily, esomeprazole 20 mg daily, and cilnidipine 10 mg daily. Folic acid was stopped owing to discontinuation of the pemetrexed-based regimen (day 4). The warfarin dose was 1.25 mg once daily. Bucolome, which has been reported to enhance the effect of warfarin [8, 9], was also used simultaneously at a dose of 300 mg once daily. The INR value was monitored closely based on a pharmacist’s suggestion, considering a possible drug interaction between warfarin and alectinib through displacement of plasma protein binding or CYP3A4. The INR increased from 2.68 to 3.15 (day 4). Although the warfarin dose was decreased to 1 mg for four days, the INR value further increased to 4.06 (day 8). Thus, warfarin and bucolome were discontinued, after which the INR value decreased to 2.75 (day 11). Warfarin was not switched with a direct oral anticoagulant because the patient had undergone valve implantation. The patient reported that nosebleeds occurred on hard nose-blowing, at least when the INR was in the therapeutic range. The patient had grade 2 diarrhea (day 10), which he treated with an over-the-counter probiotic product. Loperamide was used to treat diarrhea as needed after day 15, and Biofermin-R^®^ was administered (one tablet thrice daily) after day 18. The serum albumin level during alectinib treatment was in the range of 3.5–3.8 g/dL.

Chest computed tomography (CT) showed a partial response to alectinib two weeks after initiating treatment, and the patient was discharged from the hospital. Warfarin dosage was adjusted to maintain INR in the therapeutic range at another hospital. During an outpatient follow-up visit, the patient complained about coughing. Subsequent chest CT revealed bilateral ground-glass attenuation in the lungs. Based on the CT findings, drug-induced pneumonia caused by alectinib was suspected, which led to treatment discontinuation for 4.5 months. Pneumonia gradually improved after discontinuing alectinib. The patient was informed about the risk of interstitial pneumonia recurrence that might be caused by other ALK-TKIs.

After readmission, crizotinib was initiated at a dose of 250 mg twice daily to control tumor progression (day 0, Fig. 1b). Dexamethasone, 2 mg twice daily, and metoclopramide, 5 mg thrice daily, were administered for the first three days to prevent nausea and vomiting induced by crizotinib, which has been associated with a moderate-to-high emesis risk per the National Comprehensive Cancer Network guidelines [10]. The INR value increased from 2.57 to 3.90 (day 5). Warfarin and bucolome were discontinued, and the INR decreased to 2.93 (day 8). The patient was discharged without recurrence of interstitial pneumonia (day 14). The serum albumin level during inpatient crizotinib treatment was in the range of 3.4–3.9 g/dL. No bleeding was reported.

After progression with 6.5 months of crizotinib treatment, the patient was readmitted, and the regimen was switched to ceritinib at a dose of 750 mg once daily (day 0, Fig. 1c). The patient received metoclopramide, 5 mg thrice daily, for nausea, Biofermin-R^®^, two tablets thrice daily, and loperamide, 1 mg, as needed. On day 6, his serum amylase level increased (grade 3), after which ceritinib was stopped (INR was 2.70 on day 5). Further testing revealed that the increased amylase was an S-type amylase, which was thought to be attributable to lung carcinoma, and not a side effect of ceritinib. Grade 1 alanine aminotransferase (ALT)/aspartate aminotransferase (AST), which occurred after cessation of ceritinib 750 mg, was improved by oral administration of monoammonium glycyrrhizinate. Ceritinib was re-administered at a dose of 600 mg once daily (day 40), and the INR increased from 2.59 to 3.26 (day 47). Although the warfarin dose was decreased to 0.5 mg without stopping bucolome (day 48), the INR further increased to 3.51 (day 50). On the next day, the warfarin dose was decreased to 0.25 mg without stopping bucolome. On day 54, the INR decreased to 2.76, and liver-protective agents were prescribed against grade 1 ALT/ AST increase. Four days later, ceritinib 600 mg was discontinued because of grade 2 vomiting and poor water intake. Thereafter, the ceritinib dose was gradually decreased by 150 mg, and the INR was 3.5 two days after stopping ceritinib 150 mg once daily, which had been administered for seven days. The INR decreased to 3.22 two days after stopping warfarin and further decreased to 1.9 six days after stopping warfarin. Subsequently, ceritinib was not recommenced, and the patient did not receive any other treatment. The INR did not change thereafter. The ranges of serum albumin levels during treatment with ceritinib 750 mg, 600 mg, 450 mg, 300 mg, and 150 mg were 2.7–3.2 g/dL, 3.1–3.5 g/dL, 2.8–3.3 g/dL, 2.4–2.8 g/dL, and 2.0–2.1 g/dL, respectively. No bleeding was reported.

## Discussion and conclusions

The INR values were increased in the patient receiving warfarin and bucolome each time after initiating alectinib, crizotinib, and ceritinib treatment. Elevation of the INR value has been associated with vitamin K deficiency due to low dietary intake [11, 12], diarrhea [13], administration of antibiotics [14], or other factors, such as hepatic impairment, hypoalbuminemia [15, 16], or enhancement of the effect of warfarin because of drug interaction with other concomitant medicines.

The patient’s daily dietary intake was sufficient during treatment with alectinib or crizotinib and, at first, during treatment with ceritinib 600 mg but was too low after the maximum observed INR value. In summary, this case demonstrates that the patient’s food intake did not greatly influence the increase in INR.

Thomas et al. [17] reported elevated INR in a patient receiving warfarin and erlotinib, an epidermal growth factor receptor (EGFR)-TKI for NSCLC with *EGFR* mutations; the patient simultaneously developed “severe” diarrhea with a moderate dietary intake until erlotinib discontinuation. Our patient experienced diarrhea (grade 1–2) before alectinib initiation, after high INR values were reached during alectinib treatment, and several days before the maximum observed INR value during treatment with ceritinib 600 mg. When diarrhea developed, loperamide, which binds to 96.5% of plasma proteins and is mainly metabolized by CYP3A4 and CYP2C8, was administered. Low-grade diarrhea and occasional use of loperamide were not considered to be associated with high INR.

No antibiotics were administered during treatment with the above-mentioned ALK-TKIs. Liver function was normal during alectinib and crizotinib therapy. Grade 1 ALT/AST increase was observed after the decrease in INR during the course of treatment with ceritinib 600 mg. Basal serum albumin levels have been reported to be inversely correlated with high INR [15, 16]. Our patient had normal or near-normal serum albumin levels before the high INR values were reached during treatment with alectinib, crizotinib, and ceritinib 600 mg, but low serum albumin levels could have influenced INR control when ceritinib 150 mg was administered.

No medicines that influence the pharmacokinetics of warfarin were administered during alectinib treatment. During crizotinib treatment, the INR increased several days after the last administration of dexamethasone and metoclopramide, indicating that the use of these drugs did not coincide with the increased INR. Drugs with a high protein-binding capacity can disturb the binding between warfarin and plasma protein, causing warfarin displacement, and thereby increasing the plasma concentration of the unbound form of warfarin. This can be attributed to the small distribution volume of warfarin. In our case, displacement of plasma protein binding or competitive inhibition of metabolism by alectinib, crizotinib, and ceritinib could have increased the plasma concentration of the unbound form of warfarin, resulting in high INR. In addition, alectinib, crizotinib, and ceritinib might cause displacement of bucolome from plasma proteins, followed by displacement of warfarin or inhibition of warfarin metabolism caused by the unbound form of bucolome. *VKORC1* variants are also associated with therapeutic doses of warfarin [18].

Saito et al. [19] reported that the influence of dietary vitamin K intake on warfarin anticoagulation might be *VKORC1* genotype-dependent. In our case, a low dietary intake could have caused the INR elevation when ceritinib 150 mg was administered. Although the INR elevation was possibly based on genetic background, we did not test genetic polymorphisms of *CYP2C9* and *VKORC1* in this case. Therefore, we could not consider the relationship between the elevated INR and *VKORC1* genetic polymorphisms. Further investigation is necessary to clarify the mechanisms observed, including genetic testing and the mechanisms of these drug interactions. In this case, cause and effect relationships between increased INR values and concomitant administration of warfarin and each ALK-TKI were measured using the Drug Interaction Probability Scale (DIPS) [20]. Alectinib, crizotinib, and ceritinib were all rated as “Possible” (total score of 4) (an additional file shows this in more detail [see Supplementary Material]). In this study, ceritinib 750 mg was administered five days before the INR value increased slightly from 2.42 to 2.70. This small increase in the INR value could be attributed to the short period of treatment. After treatment with ceritinib 600 mg was initiated, the INR value increased gradually (Fig. 1c). However, while ceritinib 450 mg was administered for 14 days and 300 mg was subsequently administered for 12 days, an increase in INR value was not observed. This could be attributable to the lowering of the ceritinib dose. The INR value was not measured during the seven days of treatment with ceritinib 150 mg in the outpatient setting; however, it increased to 3.5 two days after stopping ceritinib treatment. The patient’s dietary intake was unclear because of outpatient treatment; however, the patient exhibited anorexia in the morning during the seven days. A low dietary intake may cause an increase in INR, which could depend on the dosage or length of ceritinib treatment or other factors during the use of warfarin regardless of a drug interaction. The interactions may be caused by various factors such as low dietary intake and serum albumin decrease, which might influence warfarin pharmacokinetics.

Thus, we concluded that INR values could increase in the relatively early period during treatment with alectinib, crizotinib, or ceritinib in a patient who receives concurrent treatment with warfarin and bucolome. Hence, the INR values should be monitored frequently, and warfarin dosage adjustment is needed to maintain the INR in the therapeutic range. In this case, if information related to the number of doses of warfarin that should be decreased was available, warfarin dose reduction at the beginning of coadministration could have mitigated the risk of high INR or bleeding. Moreover, this case shows that even in patients for whom direct oral anticoagulants cannot be used, coadministration of ALK-TKIs, warfarin, and bucolome could be continued without any serious bleeding events through warfarin dose adjustment.

## Supplementary Information

**Additional file 1: **Drug Interaction Probability Scale (DIPS) for Drug Interactions between ALK inhibitors and warfarin with concurrent use of bucolome: a case report. We assessed the probability of a causal relationship between a potential drug interaction and an event. Alectinib, crizotinib, and ceritinib were all rated as “Possible” (total score of 4).

## Data Availability

All data generated or analyzed during this study are included in this published article and its supplementary information file.

## Abbreviations

ALK: Anaplastic lymphoma kinase
NSCLC: Non–small-cell lung cancer
TKI: Tyrosine kinase inhibitor
CYP: Cytochrome P450
INR: International normalized ratio
CT: Computed tomography
ALT: Alanine aminotransferase
AST: Aspartate aminotransferase
EGFR: Epidermal growth factor receptor
